# Deriving and Using Descriptors of Elementary Functions in Rational Protein Design

**DOI:** 10.3389/fbinf.2021.657529

**Published:** 2021-04-13

**Authors:** Melvin Yin, Alexander Goncearenco, Igor N. Berezovsky

**Affiliations:** ^1^Bioinformatics Institute, Agency for Science, Technology, and Research (A^*^STAR), Singapore, Singapore; ^2^National Center for Biotechnology Information, National Institute of Health (NIH), Bethesda, MD, United States; ^3^Department of Biological Sciences (DBS), National University of Singapore (NUS), Singapore, Singapore

**Keywords:** protein function, protein design, elementary functional loops, elementary function, descriptor of the elementary function, DEFINED-PROTEINS software package

## Abstract

The rational design of proteins with desired functions requires a comprehensive description of the functional building blocks. The evolutionary conserved functional units constitute nature's toolbox; however, they are not readily available to protein designers. This study focuses on protein units of subdomain size that possess structural properties and amino acid residues sufficient to carry out elementary reactions in the catalytic mechanisms. The interactions within such elementary functional loops (ELFs) and the interactions with the surrounding protein scaffolds constitute the descriptor of elementary function. The computational approach to deriving descriptors directly from protein sequences and structures and applying them in rational design was implemented in a proof-of-concept DEFINED-PROTEINS software package. Once the descriptor is obtained, the ELF can be fitted into existing or novel scaffolds to obtain the desired function. For instance, the descriptor may be used to determine the necessary spatial restraints in a fragment-based grafting protocol. We illustrated the approach by applying it to well-known cases of ELFs, including phosphate-binding P-loop, diphosphate-binding glycine-rich motif, and calcium-binding EF-hand motif, which could be used to jumpstart templates for user applications. The DEFINED-PROTEINS package is available for free at https://github.com/MelvinYin/Defined_Proteins.

## Introduction

Contemporary views of the enzymatic functions are dominated by either consideration of functional domains or the catalytic active sites as their minimal structural/functional units (Marchler-Bauer et al., 2015; Finn et al., 2016; Trudeau and Tawfik, 2019). The relationships in protein function evolution, however, are far more complex than the sequence-based models can describe (Nath et al., 2014; Goncearenco and Berezovsky, 2015; Aziz et al., 2016; Romero Romero et al., 2016; Berezovsky et al., 2017a). In many cases, the closely related structurally similar folds can carry completely different biochemical functions, while, on the other hand, the same function can be performed by many different protein folds (Goncearenco and Berezovsky, 2015; Berezovsky, 2019). According to the Enzyme Commission number (EC) nomenclature (Bairoch, 2000), the current number of enzymatic functions reaches up to 5,000. The number of underlying biochemical mechanisms (Holliday et al., 2012), however, does not exceed 500; moreover, the number of elementary chemical reactions (Holliday et al., 2005) is less than 50. The differences in the order of magnitude between the numbers of enzymatic functions, biochemical mechanisms, and elementary chemical reactions prompt one to consider the biochemical function as a combination of elementary ones. Also, the domains themselves had to evolve from some primitive forms (Berezovsky, 2003, 2019; Nath et al., 2014; Goncearenco and Berezovsky, 2015; Aziz et al., 2016; Romero Romero et al., 2016, 2018; Berezovsky et al., 2017a,b). It has been hypothesized that the first group of enzymatic domains emerged as combinations of prebiotic (Romero Romero et al., 2016) ring-like peptides with simple chemical transformation (Trifonov et al., 2001; Goncearenco and Berezovsky, 2015; Berezovsky et al., 2017a; Berezovsky, 2019). Closed loops of preferential 25 to 35-amino acid residue size, a universal basic element of soluble proteins, are the descendants of the prebiotic peptides (Berezovsky et al., 2000, 2017a,b; Berezovsky, 2003) determined by the polymer nature of polypeptide chains (Yamakawa and Stockmayer, 1972; Shimada and Yamakawa, 1984; Berezovsky et al., 2000, 2017a; Orevi et al., 2013; Jacob et al., 2018).

Previous studies showed that biochemical functions can be represented as a combination of the elementary ones, provided by the elementary functional loops (EFLs), which are closed loops with specific signatures that perform elementary steps of biochemical transformations (Goncearenco and Berezovsky, 2010, 2011, 2012, 2015). We suggest that EFLs can be considered as potential elementary units in the design of biochemical functions, which requires an exhaustive description of their characteristics that are important for building the required catalytic site in the environment of the particular protein fold. Even though EFLs perform only elementary steps of the biochemical reactions, the relationship between their sequences, structures, and functions is complex. For example, CxxC motifs (Goncearenco and Berezovsky, 2011) are known to be involved in a variety of functions, such as metal or metal-containing cofactor binding or redox reactions. Consequently, structures of the EFLs containing this signature in many folds differ significantly (Goncearenco and Berezovsky, 2012; Zheng et al., 2016), depending on both the structural environment in the protein and its overall biochemical function. The interactions between the EFL and the substrate and between the EFL and the rest of the structure will also depend on the fold and its function (Berezovsky, 2019). Therefore, the descriptor of EFL has to capture structure- and function-dependent interaction propensities.

The protein design adventure (Das and Baker, 2008) started more than 50 years ago from the general protein folding problem (Dill and MacCallum, 2012) formulated in terms of polymer and statistical physics (Shakhnovich and Gutin, 1993) of biomolecules (Sali et al., 1994; Shakhnovich, 2006), in terms of statistical predictions of structures from the sequence (Sippl, 1990; Crippen, 1996), and as an inverse protein-folding problem (Rooman et al., 1990; Bowie et al., 1991; Rooman and Wodak, 1995) of finding the sequence that can be threaded into the certain fold structure. Current progress in the evolutionary-inspired fragment-based (Hocker, 2014) and *de novo* (Huang et al., 2016a; Silva et al., 2019) design is described in several original works (Brunette et al., 2015) and reviews (Lechner et al., 2018; Baker, 2019; Berezovsky, 2019). It is further facilitated by advances in machine learning and artificial intelligence, as well as by the quality and quantity of high-throughput sequence and structural data available, leading to significant improvements in the performance of computational approaches (Senior et al., 2020). Despite significant progress in the computational design of protein structures, the journey toward solving the great challenge of the *de novo* design of protein functions is, as of yet, at its very beginning (Huang et al., 2016a; Lechner et al., 2018; Baker, 2019; Berezovsky, 2019). Although the repertoire of conserved continuous functional units is available on the sequence level, a more comprehensive characterization is required to define spatial and interaction restraints (Berezovsky, 2019). The computational framework presented in this study facilitates the derivation of the descriptor of elementary function and conceptualizes the objective function for protein engineering and design applications using the descriptor. It merges structure, sequence, and interaction features important for defining elementary functions on a residue level. In protein design, descriptors of elementary functional units serve as off-the-shelf building blocks, for instance, in protein grafting, while the objective function optimizes the choice of such building blocks from a library, considering their geometry and interactions with the protein scaffold, particularly in the key catalytic or binding residues.

We illustrate this approach by calculating the descriptors for three ubiquitous ELFs: the calcium-binding EF-hand, the phosphate-binding in mononucleotide-containing ligands, and the phosphate-binding in dinucleotide-containing ligands, such as ATP, nicotinamide–adenine–dinucleotide (NAD), and NAD phosphate (NADP), in a variety of structural scaffolds. We also model a hypothetical grafting experiment by swapping the EFLs and EFL-derived chimeras among the scaffolds.

## Materials and Methods

The proof-of-concept computational framework is aimed at, first, derivation of the descriptor of elementary function and, second, application of descriptors to design proteins with desired structures and functions. A descriptor represents a set of characteristics of the EFL (Goncearenco and Berezovsky, 2010, 2011, 2012; Berezovsky et al., 2017a), including the position-specific information on the sequence, and several structural features encoded as probabilistic distributions (Berezovsky, 2019). An elementary function is defined as the smallest structural unit sufficient to carry out an elementary reaction in a biochemical transformation. Depending on the protein engineering task, it may be needed to introduce or replace an elementary function in an existing protein of interest or build and design a protein with the required structure and function *de novo* (Berezovsky, 2019). The flowchart in Supplementary Figure 1 illustrates the sequence of steps described below.

### Deriving the Descriptor

Motivated by the biophysical constraints of the polypeptide chain (Goncearenco and Berezovsky, 2010, 2011; Berezovsky et al., 2017a; Berezovsky, 2019), the procedure starts from 30-residue long seed sequence fragments of the functional loops represented by a gapless multiple sequence alignment and a position-specific scoring matrix (PSSM) profile. The sequence profile is then iteratively scanned against the non-redundant UniRef database (Hunter et al., 2009) with an expectation–maximization (EM)-like algorithm (Goncearenco and Berezovsky, 2010, 2011) converging to an expanded sequence profile of the descriptor with a functional signature (Berezovsky et al., 2003a,b). The corresponding structures characterizing the functional loop are then obtained by looking for profile matches against the sequences in the Protein Data Bank (Berman et al., 2000; wwPDB consortium, 2019), followed by extraction and encoding of the structural feature in the form of parametrized probability distribution functions. Structural and functional annotations are extracted from the corresponding databases, such as Uniprot, MaCiE, and Conserved Domains Database (CDD) (Bairoch, 2000; Andreini et al., 2009; Fischer et al., 2010; Holliday et al., 2012; Marchler-Bauer et al., 2013; Akiva et al., 2014; Furnham et al., 2014). The structural features include dihedral angles, Van der Waals (VdW) interactions, and hydrogen bonds (H-bonds). Intra-EFL interactions and interactions between the functional loop and the rest of the structure are encoded separately. Thus, a descriptor contains information about the immediate environment of the functional loop in all protein scaffolds and enzymatic functions where it was encountered.

### Objective Function for Protein Engineering and Design

In protein engineering and *de novo* design, the descriptors of elementary function need to be integrated into a given structural scaffold. Once the elementary function that is required to be incorporated into the protein is selected, the sequence, structure, and interactions that would fit best into the scaffold have to be determined. The objective function scores how well a given structural loop fits in and should be maximized to obtain the best matching implementation of the descriptor with an assumption that the native structure has the best fit. Essentially, the score represents the joint likelihood of all amino acid positions in the grafted loop with respect to distributions parametrized in the descriptor with N-residue positions and M features: $\;F=\overset{\text{N}}{\underset{\text{i}}{\sum }}\overset{\text{M}}{\underset{\text{j}}{\sum }}{W_{\text{i}}^{P}W}_{\text{ij}}^{\text{F}}S_{\text{ij}}$. The weight that is given to a residue position ${\text{W}}_{\text{i}}^{\text{P}}$ reflects the relative degree of conservation of features in each position: ${\text{W}}_{\text{i}}^{\text{P}}=\frac{\overset{\text{M}}{\underset{\text{j}=0}{\sum }}{\text{W}}_{\text{ij}}^{\text{F}}}{\overset{\text{N}}{\underset{\text{i}=0}{\sum }}\overset{\text{M}}{\underset{\text{j}=0}{\sum }}{\text{W}}_{\text{ij}}^{\text{F}}}\;$. Descriptor features *j* enumerate along a sequence signature (*j* = *a*), dihedral angles (*j* = *d*), H-bonds (*j* = *h*), and vdW interactions (*j* = *v*), with the corresponding scores *S*_ij_ and weights *w*_ij_. Score *S*_*i,a*_ is the log-odds score for amino acid substitution according to BLOSUM62 (Henikoff and Henikoff, 1993). The weight of the residue feature is given by the relative frequency of the two most frequent residues in the sequence profile ${\text{W}}_{\text{i},\text{a }}^{\text{F}}=\frac{\overset{2}{\underset{\text{k}}{\sum }}\left(\text{argma}x_{\text{k}}{\text{w}}_{\text{k}}\right)}{\overset{20}{\sum }{\text{w}}_{\text{k}}}$, where w_k_ is amino acid frequency. Dihedral angles are first clustered as two-dimensional vector quantities using the EM algorithm as implemented in scikit-learn, and their weights and fitting scores are derived from parameters in the trained model with the following equation:

${\text{S}}_{\text{i},\text{d}}=1-\mathrm{erf}\left(\sum {\text{D}}^{\text{T}}\text{CD}\right)$, where *erf* refers to the error function, D = X - Y is the displacement of the compared dihedral angle points, normalized by the median of all points in the descriptor, expressed as phi-psi angles, and *C* is a matrix proportional to the degree of confidence that the point belongs to the distribution. Given the precision matrix Λ=σ^−2^ and posterior probability matrix *P* obtained from the clustered model, C = Λ P.

The isotropic VdW distribution score, S_i,v_, is based on counting the number of VdW contacts with non-hydrogen atoms within the 5-Å radius. Hydrogen bonds are additionally split into acceptors and donors: S_i,hA_ and S_i,hD_, respectively. They are scalar quantities and are measured as the number of donor H-bonds present at the residue position. For H-bonds, the effective radius is 3.5 Å. The score is the ratio between the absolute difference in the number of bonds (δ) to the higher number of contacts for either structure (VdW interactions and H-bonds): ${\text{S}}_{\text{i},\;\left[\text{d},\text{v},\text{hA},\text{hD}\right]\;}=\frac{\text{abs}\left({\text{δ}}_{\text{x}}-\delta_{y}\right)}{\text{max}\left({\text{δ}}_{\text{x}},\;\delta_{\text{y}}\right)}$. The weight of the feature is the SD of the spread of a half-normal distribution fitted onto the data ${\text{W}}_{\text{i }\left[\text{b},\text{c},\text{d},\text{e}\right]}^{\text{F}}=\sigma$. Each feature weight is further tuned by an empirically derived scalar factor to account for differences in the spread of absolute values returned by the scoring functions.

## Results

### Deriving the Descriptors of EFs

**Figure 1:**
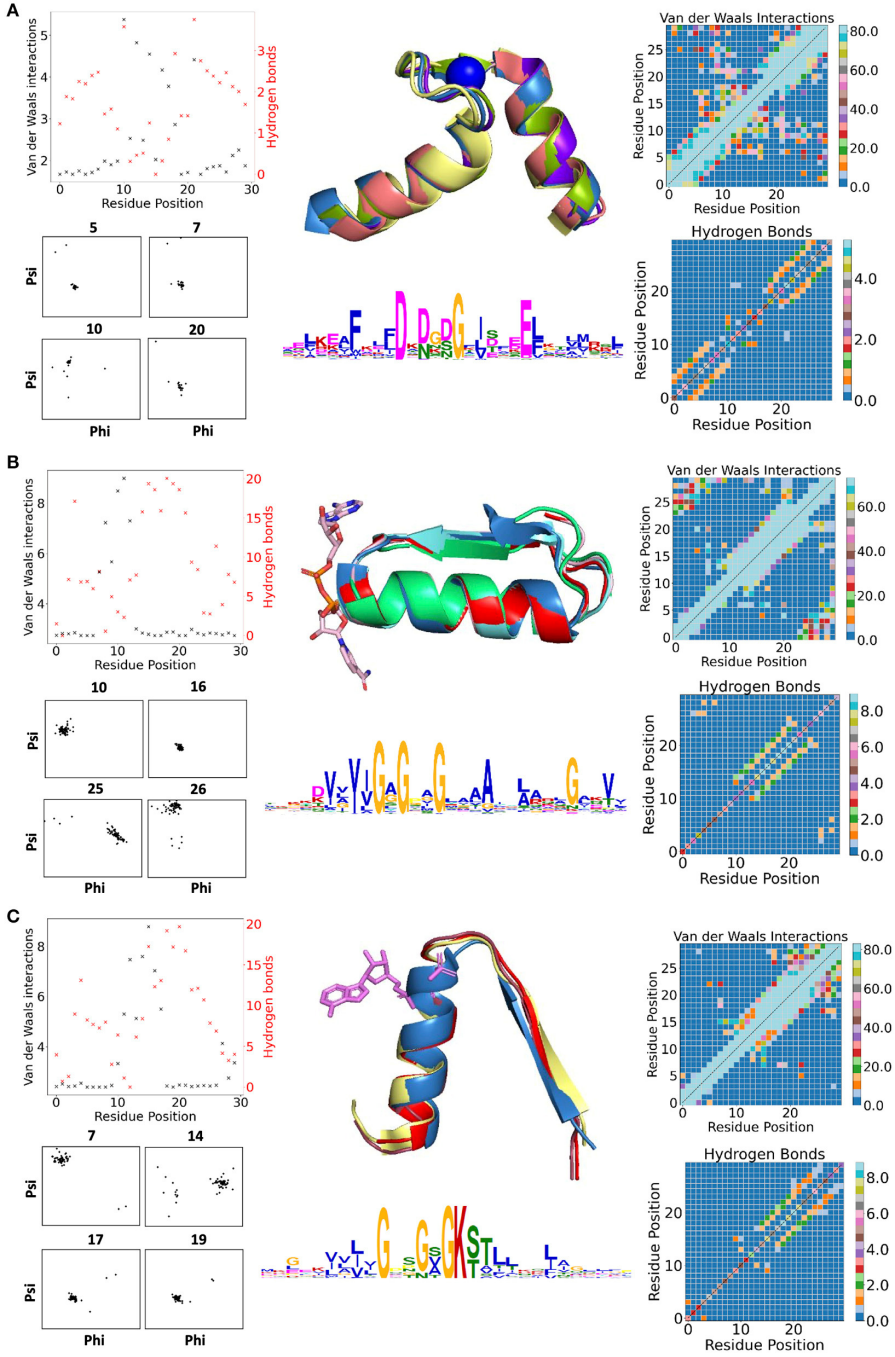
**(A)** Descriptors for Ca^2+^-binding helix-loop-helix EF-hand motif (Gifford et al., 2007), **(B)** phosphate-binding loop in dinucleotide-containing ligands, and **(C)** phosphate-binding P-loop. Charts in the left column show per-residues numbers of Van der Waals (VdW) interactions and hydrogen bonds (H-bonds); contact maps in the right column show the total number of VdW contacts in H-bonds in all structures used for the derivation of corresponding descriptors. The central column contains examples of structures used for derivation of corresponding descriptors (represented in the form of structurally aligned segments) along with the logos representing the position-specific matrices of derived descriptors. The numbering of residues is sequential and follows positions in the logo.

Figure 1 contains examples of some of the structural features for three descriptors: the calcium-binding Ca^2+^-binding helix–loop–helix EF-hand motif (Gifford et al., 2007) (with the characteristic signature DxDxD, Figure 1A), the glycine-rich motif of the phosphate-binding in dinucleotide-containing ligands (with the characteristic signature GxGxxG, Figure 1B), and the phosphate-binding P-loop in nucleotide-containing ligands (with the characteristic signature GxxGxG, Figure 1C). For EF-hand (Gifford et al., 2007), dihedral angles on amino acid positions before and after the calcium-binding sites form tight clusters, i.e., the backbone is structurally conserved. Donor-acceptor hydrogen bond pairs spaced three to four residue positions apart are also present in the first and last 10 residues of the EF-section (Figure 1A), representing, together with conserved dihedral angles, two α-helices in the structural motif. Distributions of VdW contacts in the EF-hand motif show a large proportion of the conserved intrinsic contacts flanking the α-helices, whereas the central flexible link between them forms vital external contacts with the rest of the fold. The same patterns of the internal contacts are observed in the second halves of the GxGxxG and GxxGxG motif structures also containing the α-helices. The β-strands in these loops, as expected, interact more strongly with the rest of the structure via VdW interactions (Supplementary Figure 2) and H-bonds (Supplementary Figures 3, 4). In addition to the interactions with ligands, the substrate-binding residues in the descriptor are characterized by multiple interactions with the fold.

### Swapping the EFLs Within the Same Structural/Functional Context

The objective function was calibrated and exemplified on three motifs: (i) the EF-hand from *Bos taurus* calcium-binding protein structure (PDBID 1A29) with the Ca^2+^ ligand; (ii) the phosphate-binding motif (GxGxxG) from *Equus caballus* oxidoreductase (PDBID 1A71) with dinucleotide-containing NAD ligand; and (iii) the phosphate-binding motif (GxxGxG) from *Methanocaldococcus jannaschii* ABC transporter (PDBID 1G6H) with nucleotide-containing an ADP ligand. These motifs were replaced with the realization of the corresponding descriptors (see Supplementary Figure 5 and explanations in the figure caption).

Supplementary Figure 6 illustrates how objective function can be used to obtain the best descriptor realization in the corresponding engineering or design tasks. First, a segment of seven consecutive residues (half of the typical functional signature; Berezovsky et al., 2017a) with the highest cumulative fitting score is determined. Then, this segment is extended with residues that contribute the highest scores to the fitting function. There might be insertions/deletions in the descriptor realization, or the reference could be undefined as in the case of missing data, with disordered or structurally unresolved regions in the query structure. In such a case, the algorithm first scores all valid residue positions and finds the appropriate segments to merge if the best match does not come from a singular structure. Next, adjacent segments are extended, possibly from both ends in the case of a gap, to fill in the uncharacterized positions. This returns the best-effort re-engineered structure that partially relies on the input structure and fills in the rest, where data are missing. Supplementary Figure 6 exemplifies the case of descriptor realization that builds the functional loops out of two segments, providing the most optimal score.

### Swapping EFLs Between Different Functions and Structures in the Cross-Validation Experiment

To assess the robustness of the derived descriptors and versatility of the fitting function, we selected seven structures with the phosphate-binding functionality, but of different folds, origins, and ligands: NADP-binding *Thermotoga maritima* lactate dehydrogenase (1A5Z) and *Methylobacterium extorquens AM1* methylene-tetrahydromethanopterin dehydrogenase (1LUA), AMP-binding *Escherichia coli* MoeB-MoaD protein complex (1JWB), flavin–adenine dinucleotide (FAD)-binding *Klebsiella pneumoniae* udp-galactopyranose mutase (2BI7) and *Thermus thermophilus GidA-related protein*, and both binding sites for FAD and NADP in *E. coli* CoA reductase (1PS9) and human dihydrolipoamide dehydrogenase (1ZMC).

**Figure 2:**
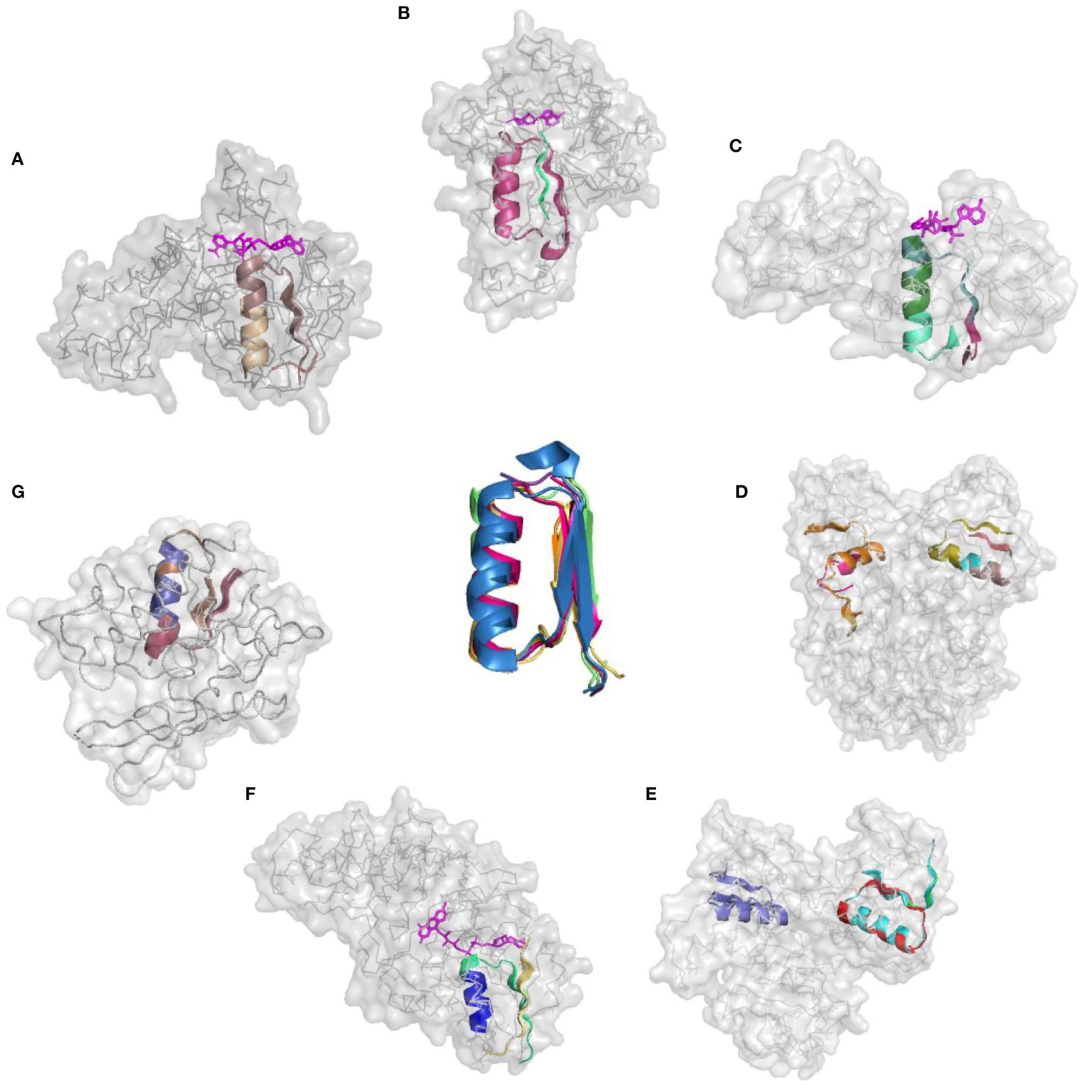
Realizations of the descriptor of Gly-rich signature of the phosphate binding in dinucleotide-containing ligands. **(A–G)** PDB IDs of corresponding structures are: 1A5Z, 1JWB, 1LUA, 1PS9, 1ZMC, 2BI7, and 2CUL, respectively.

The donor functional loop that had to be transplanted is an elementary function of phosphate binding in dinucleotide-containing ligands (GxGxxG). We derived its descriptor from the dataset that excluded the aforementioned structures. Seven proteins representing variations in the structure and sequences of the phosphate-binding functional loops (both in nucleotide- and dinucleotide-containing ligands) were used as the targets or acceptors for the EFL-replacement procedure, using the above descriptor. Figure 2 illustrates the fit between the re-engineered functional loop into the original structure, which binds various ligands, including AMP, FAD, and NAD(P). Additionally, recombinant functional loops can be built from the best matching segments of multiple structures. Taking 1PS9 structure as an example, its NADP binding site is replaced with the loops from structures 1KF6 from *E. coli* quinol-fumarate reductase; 1VRP from *C. sarcosine* oxidase; 6GNC from *Clostridium acetobutylicum* thioredoxin reductase; and 5ER0 from *Lactobacillus* oxidase. All of these structures contain FAD-binding sites replacing the original NADP-binding site.

### Proof-of-Principle Grafting Experiment Using Descriptors of the Phosphate-Binding EFLs With GxGxxG and GxxGxG Signatures

**Figure 3:**
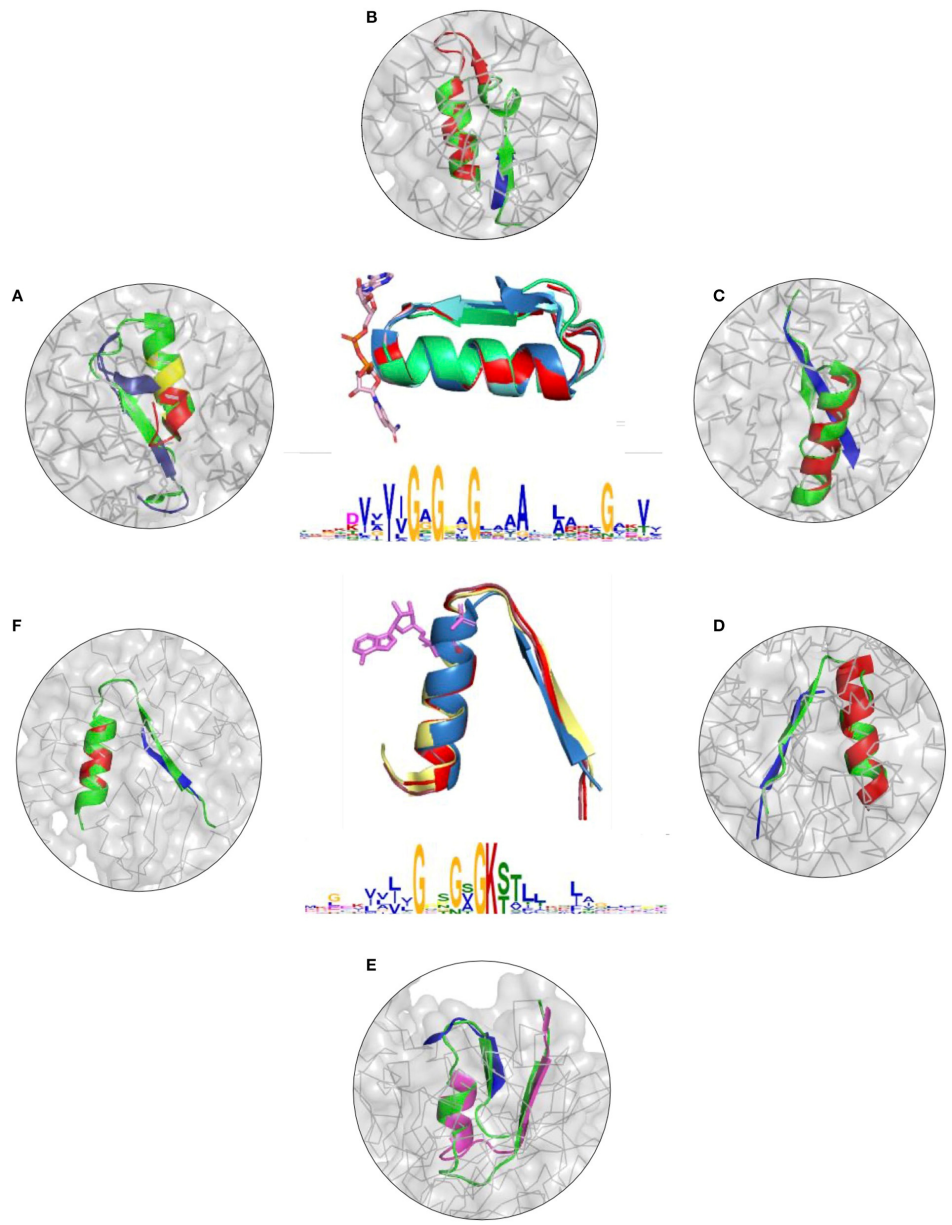
**(A–F)** Cross-grafting of functional loops of the phosphate binding in dinucleotide (GxGxxG) and nucleotide-containing (GxxGxG) ligands. **(A–C)** Grafting of the phosphate-binding signature in dinucleotide-containing ligands (GxGxxG) in proteins with P-loop (GxxGxG) elementary function; recombinant realizations of descriptors are shown in proteins with PDB IDs: 1H5Y, 1BWV, and 1O5K. **(D–F)** Recombinant realizations of the descriptor of P-loop (GxxGxG) elementary function in proteins (1SKY, 1II2, and 1NI3, respectively) with elementary functional loops of the phosphate-binding in dinucleotide-containing ligands (GxGxxG). The original structures are shown in green.

To further illustrate the utility of descriptors of elementary functions and the potential of the DEFINED-PROTEINS package in protein design applications, we set up initial conditions for the grafting procedure where we replaced the original functional loop in a given protein with the non-native one representing a different elementary function. We recruited two distinct, but not opposite, elementary functions, for which we have already derived descriptors: binding of the phosphate in dinucleotide-(GxGxxG) and nucleotide-containing P-loop (GxxGxG) ligands (Zheng et al., 2016). Thereby, we cross-grafted nucleotide-binding function into protein folds with native dinucleotide-ligand binding (Figures 3A–C and Supplementary Figures 7A–C), and cross-grafted dinucleotide-binding function into folds with the native mononucleotide binding ability (Figures 3D–F and Supplementary Figures 7D–F), respectively. Although both are elementary functions of the phosphate binding, the latter belong to different dinucleotide- and nucleotide-containing ligands that determine the corresponding diversity of protein functions that these elementary functions evolved into.

The signatures of EFLs and the most frequent interactions with distinct parts of ligands were discussed elsewhere (Berezovsky, 2019), showing both conservatism and diversity depending on the position in the functional loop. From the structural perspective, both EFLs have similar secondary structures of β-turn-α-helix composition and architecture. The latter results in a high number of intrinsic H-bonds and VdW contacts in its second α-helical part, while β-strand elements of both loops form more contacts with the surrounding structure. At the same time, the dinucleotide-binding-GxGxxG-loop is a more compact structure by itself, with more intrinsic contacts between its α and β elements. We replaced the original functional segments with recombinant (Figure 3) and deconvoluted single-loop (Supplementary Figure 7) functional loops sampled from the corresponding descriptors. In all of the cases presented in Figure 3 and Supplementary Figure 7 replacements were done with the highest-scoring matches.

**Figure 4:**
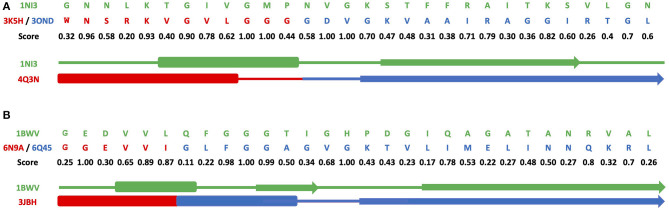
Alignments for recombinant cross-grafting of the phosphate-binding loop in dinucleotide (GxGxxG) and nucleotide-containing (GxxGxG) ligands. **(A)** Replacement of the P-loop elementary function in 1NI3 and **(B)** the phosphate-binding signature in dinucleotide-containing ligands in 1BWV. The thick, thin, and arrow sections represent the beta sheet, turn section, and alpha helix secondary structures, respectively.

Figures 3A–C and Supplementary Figures 7A–C show results of grafting of the P-loop (GxxGxG) descriptor in places of Gly-rich signature (GxGxxG) of the phosphate-binding in dinucleotide-containing ligands. While obtaining a good match between the original and replacement loops in both unblended single-loop and recombinant cases, the latter was allowed to obtain a better per-residue fit between the original and replacement loops. Recombinant loop replacements consist of several fragments (typically, two to three), covering most of the loop (see examples in Figure 4 and Supplementary Figure 8) with higher scores in the functional positions at the expense of non-function-bearing positions in the secondary structure elements. It agrees with less conserved dihedral angles in the turn segments of the loops that contain functional signatures, which also result in different angles between the secondary structure elements of the loop and difference in the intra-loop contacts in GxxGxG and GxGxxG loops.

Figure 4 and Supplementary Figure 8 show a comparison of secondary structure alignments in several examples of the single-loop and recombinant replacements. The secondary structure is affected by the environment (Minor and Kim, 1996), pointing to the need for adjustments and optimization in length and location after the original descriptor realization is placed instead of the natural ELF. Overall, examples of structural replacements (Figures 3 and Supplementary Figure 7) and alignments (Figure 4 and Supplementary Figure 8) show that realizations of descriptors can be used as a starting point for further optimization of positions and interactions involving functional residues and structures of elementary functional loops to obtain required modifications/design in the context of new protein structures and functions.

It is important to note that, in general, scores obtained with the objective function depend on the sequence/structure characteristics of the elementary function and the type of procedure in which the descriptor is used. The major contributors to the objective function score are sequence conservation and dihedral angles, with weights 0.62/0.52 and 0.3/0.38 for the GxxGxG/GxGxxG signatures, respectively. Weights for VdW interactions and H-bonds are smaller (see Supplementary Table 1 legend for details): the VdW interactions are weak though omnipresent, whereas H-bonds between the loops and rest of the folds are rare, making the weights of both smaller. Nevertheless, contributions of all characteristics to the weight for each individual position are calculated as a sum of their weights normalized by the total feature weights across all residue positions. The score of the reference state for the descriptor, which can be indicative of the latter and can be used as a guideline in design, should be obtained for each descriptor. The straightforward way is to perform the cross-validation experiment, which assesses the robustness of the descriptor and provides the score that can be used as a ground state score. Supplementary Table 1B contains averaged scores obtained in the cross-validation experiment (Figure 2), which can be used as a reference for the engineering and design of corresponding descriptors in other folds. As an example, Supplementary Table 2B shows scores in case of cross-grafting of descriptors of the phosphate-binding signatures in the nucleotide- (GxxGxG) and dinucleotide-containing (GxGxxG) ligands, revealing the deviation from scores in cross-validation that can further increase in case of *de novo* design of functions based on the descriptors. The utility of averaged (Supplementary Tables 1A, 2A) and per-residue scores is in the information on the relative conservatism (importance) of positions in the descriptor and of the overall match between its realization and the rest of the fold and its capacity to contribute to engineered/designed function. For example, depending on the requirements on the interactions within the ELF and between the loop and the rest of the fold, H-bonds can be introduced in positions with a conservatism level allowing to do so, but the same holds for changing other characteristics.

## Discussion

There are two major tasks, engineering/modification and *de novo* design, in which descriptors of elementary functions can be used (Berezovsky, 2019). The former is a modification of the natural protein function by replacing one or several natural functional loops (elementary functions) in the protein with other elementary function(s) encoded in the corresponding descriptor(s). The goal of this engineering effort can be to change a substrate specificity to modify a biochemical function and/or mechanism of the enzyme (Babbitt et al., 1996; Pegg et al., 2006; Berezovsky, 2019). The quest on *de novo* design of the protein with required function can be set by providing the sequence, structure, or the sequence–structure combination (Leaver-Fay et al., 2011; Berezovsky, 2019). The specific task that DEFINED-PROTEINS presented in this study addresses the finding of a structural segment according to the functional descriptor, which will fit best into the original fold, providing required functional and stabilizing interactions with the rest of the fold. Ultimately, DEFINED-PROTEINS is aiming to build catalytic sites with desired activity and interactions while maintaining the overall fold structure and stability.

Steady progress in the computational design of new topologies and functions (Huang et al., 2014, 2016b; King et al., 2015) and even a stronger drive toward *de novo* protein design (Huang et al., 2016a; Lechner et al., 2018; Baker, 2019; Silva et al., 2019) prompt the use of the basic units that would possess all traits determined by the polymer nature of proteins (Yamakawa and Stockmayer, 1972; Shimada and Yamakawa, 1984; Berezovsky et al., 2000, 2017a; Orevi et al., 2013; Jacob et al., 2018), their evolutionary history, and requirements on the structural stability and dynamics, as well as show the required functional activity (Berezovsky, 2019; Romero-Romero et al., 2021). The concept of the elementary function standard/descriptor allows one to consider individual steps of biochemical functions provided by physics-based and evolutionary selected ELFs (Berezovsky, 2019). An exhaustive description of elementary functions including all sequence, structure, and functional information that can be used in the design of new biochemical functions consisting of different combinations of elementary ones. Ultimately, the set of descriptors should, as exhaustively as possible, represent the diversity of sequence signatures that perform this elementary function, as well as the diversity of their structural implementations in different protein folds. In the combinations of descriptors into the desired biochemical function, the observables of the parameters of descriptors will be the result of the interference between their distributions and the type of the final structure that carries the function, the type of the overall transformation, interactions with other descriptors involved in the construction, and interactions with the substrate. Ideally, it should be possible to use descriptors of elementary functional units to build the geometry, the set of interactions, and the environment necessary for specified biochemical function. Then, the library of these units is supposed to be used in design efforts such as, the Rosetta enzyme design protocol (Leaver-Fay et al., 2011), coupled with which the placement and refinement of a DEFINED-PROTEINS functional unit into a designable scaffold, which would include modeling of intra-loop and loop-fold interactions and energy minimization toward stable structure with required dynamics, would be enabled.

## Conclusions

The DEFINED-PROTEINS is a proof-of-concept implementation that provides the tools to: (i) derive descriptor of elementary functions of interest directly from protein structures and (ii) apply descriptors in computational engineering and design. The software is available as a Python package that allows for integration in custom protein design workflows. We exemplified the derivation of the descriptor on three elementary functions: the calcium-binding EF-hand (Gifford et al., 2007), the glycine-rich motif of the phosphate-binding in dinucleotide-containing ligands, and the mononucleotide phosphate-binding P-loop. We also assessed the robustness of derived descriptors and the objective function by replacing phosphate-binding EFLs in seven proteins with different functions derived on the set of proteins excluding the above seven proteins. Finally, we demonstrated a proof-of-principle grafting experiment by cross-replacing functional loops between P-loop containing proteins and those with the elementary function of the phosphate-binding in dinucleotide-containing ligands.

## Data Availability Statement

The software was built primarily using Python3 (http://www.python.org), C with an optional MPI requirement, and C++. Django (https://djangoproject.com) was used as the web framework with embedded interactive Bokeh plots (https://bokeh.org/). Docker container is available for OS-independent deployment. The software is BSD-licensed.

## Author Contributions

IB: conceptualization, supervision, project administration, and funding acquisition. AG and IB: methodology. AG, MY, and IB: investigation, formal analysis, and writing—review and editing. MY: software and visualization. MY and IB: writing—original draft. All authors contributed to the article and approved the submitted version.

## Conflict of Interest

The authors declare that the research was conducted in the absence of any commercial or financial relationships that could be construed as a potential conflict of interest.
