# Supplementary material for: Deriving and Using Descriptors of Elementary Functions in Rational Protein Design
Source: Front Bioinform. 2021 Apr 13;1:657529. doi: 10.3389/fbinf.2021.657529 (PMC9581014; doi:10.3389/fbinf.2021.657529)
Supplement: Supplementary file 2 [file Table_2.docx]

**Table S2. The objective function scores obtained in the cross-validation experiment (see also Figure 3 and S7).** **A.** Per-residue scores. **B.** Averaged (over all positions) score.

**A.**

| **PDB_ID** | **1H5Y** | **1BWV** | **1O5K** | **1SKY** | **1II2** | **1NI3** |
| --- | --- | --- | --- | --- | --- | --- |
| **Position** | **Score** | | | | | |
| **1** | 0.42 | 0.42 | 0.63 | 0.9 | 0.42 | 0.2 |
| **2** | 0.54 | 0.18 | 0.2 | 0.6 | 0.12 | 0.25 |
| **3** | 0.18 | 0.25 | 0.92 | 0.25 | 0.37 | 0.65 |
| **4** | 0.9 | 0.37 | 0.95 | 0.35 | 0.45 | 0.6 |
| **5** | 0.5 | 0.7 | 0.6 | 0.8 | 0.16 | 0.45 |
| **6** | 0.72 | 0.8 | 0.15 | 0.76 | 0.76 | 0.72 |
| **7** | 0.64 | 0.82 | 0.7 | 0.35 | 0.54 | 0.75 |
| **8** | 0.35 | 0.5 | 0.2 | 0.4 | 0.2 | 0.4 |
| **9** | 0.3 | 0.35 | 0.45 | 0.62 | 0.22 | 0.22 |
| **10** | 0.45 | 0.39 | 0.38 | 0.68 | 0.2 | 0.34 |
| **11** | 0.6 | 0.75 | 0.7 | 0.52 | 0.43 | 0.7 |
| **12** | 0.35 | 0.72 | 0.4 | 0.58 | 0.65 | 0.84 |
| **13** | 0.18 | 0.61 | 0.65 | 0.38 | 0.2 | 0.16 |
| **14** | 0.14 | 0.62 | 0.3 | 0.34 | 0.3 | 0.25 |
| **15** | 0.94 | 0.71 | 0.84 | 0.63 | 0.4 | 0.74 |
| **16** | 0.5 | 0.25 | 0.79 | 0.62 | 0.37 | 0.45 |
| **17** | 0.72 | 0.84 | 0.52 | 0.41 | 0.5 | 0.52 |
| **18** | 0.64 | 0.81 | 0.64 | 0.85 | 0.4 | 0.58 |
| **19** | 0.5 | 0.2 | 0.5 | 0.45 | 0.3 | 0.5 |
| **20** | 0.16 | 0.4 | 0.26 | 0.37 | 0.6 | 0.71 |
| **21** | 0.56 | 0.42 | 0.34 | 0.1 | 0.4 | 0.54 |
| **22** | 0.35 | 0.46 | 0.17 | 0.64 | 0.45 | 0.7 |
| **23** | 0.6 | 0.55 | 0.8 | 0.4 | 0.34 | 0.56 |
| **24** | 0.2 | 0.32 | 0.4 | 0.62 | 0.7 | 0.64 |
| **25** | 0.28 | 0.3 | 0.41 | 0.61 | 0.39 | 0.43 |
| **26** | 0.4 | 0.53 | 0.25 | 0.73 | 0.62 | 0.3 |
| **27** | 0.8 | 0.74 | 0.82 | 0.56 | 0.3 | 0.65 |
| **28** | 0.6 | 0.56 | 0.85 | 0.74 | 0.49 | 0.67 |
| **29** | 0.84 | 0.41 | 0.76 | 0.2 | 0.65 | 0.25 |
| **30** | 0.5 | 0.4 | 0.9 | 0.26 | 0.25 | 0.26 |

**B.**

| **Averaged over positions objective function scores in the garfting experiment** | | | |
| --- | --- | --- | --- |
| **PDB ID** | **Original Motif** | **Replacement Motif** | **Average Position Score** |
| **1H5Y** | GxGxxG | GxxGxG | 0.49 |
| **1BWV** | GxGxxG | GxxGxG | 0.51 |
| **1O5K** | GxGxxG | GxxGxG | 0.55 |
| **1SKY** | GxxGxG | GxGxxG | 0.52 |
| **1II2** | GxxGxG | GxGxxG | 0.41 |
| **1NI3** | GxxGxG | GxGxxG | 0.50 |
