# Supplementary material for: Deriving and Using Descriptors of Elementary Functions in Rational Protein Design
Source: Front Bioinform. 2021 Apr 13;1:657529. doi: 10.3389/fbinf.2021.657529 (PMC9581014; doi:10.3389/fbinf.2021.657529)

Figure S1. Workflow of the DEFINED-PROTEINS (Descriptor of Function IN Engineering and Design - PROTEINS) software package.

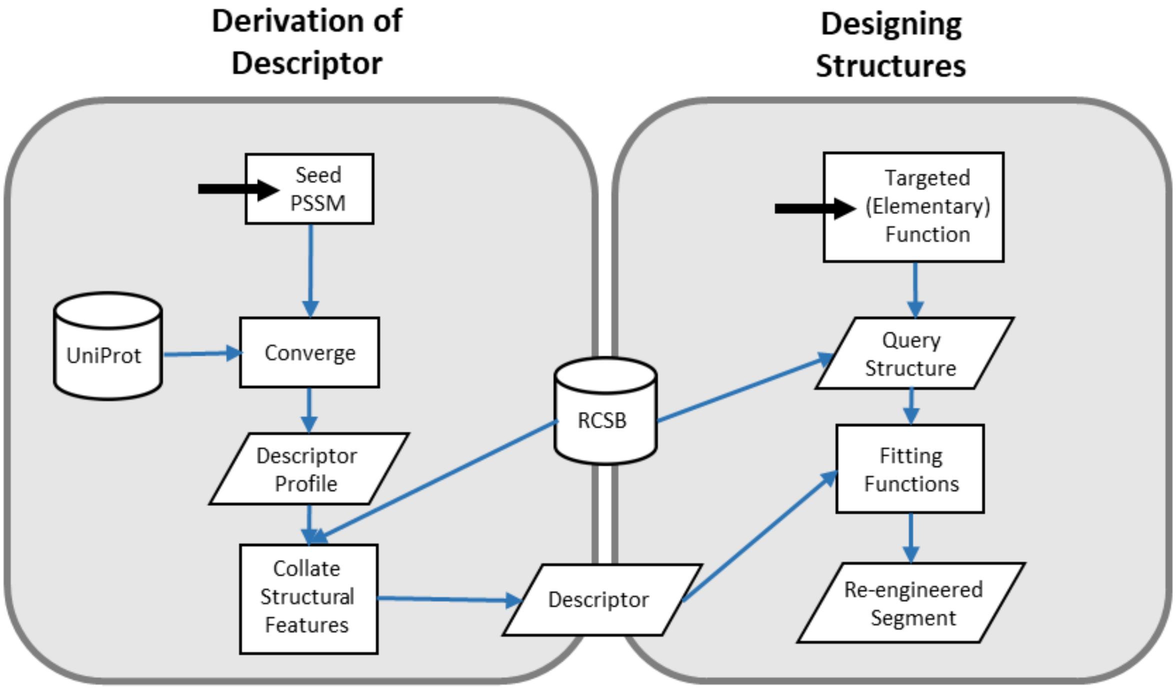

Supplement: Supplementary file 3 [file Image_1.PDF]
