# Supplementary material for: Deriving and Using Descriptors of Elementary Functions in Rational Protein Design
Source: Front Bioinform. 2021 Apr 13;1:657529. doi: 10.3389/fbinf.2021.657529 (PMC9581014; doi:10.3389/fbinf.2021.657529)
Supplement: Supplementary file 4 [file Image_2.PDF]

Figure S2: VdW interactions at 5A isotropic shell radius for DxDxD (first row), GxGxxG (second row), and GxxGxG (third row) signatures of corresponding elementary functions (see main text for designation of elementary functions). The total number of interactions (A) is the sum of the internal (B) and external (C) interactions. The GxGxxG and GxxGxG loops have a similar secondary structure configuration, namely a beta sheet turning into an alpha helix, and their VdW interaction distributions illustrate that similarity. The turn occurs at around residue position 10 for both.

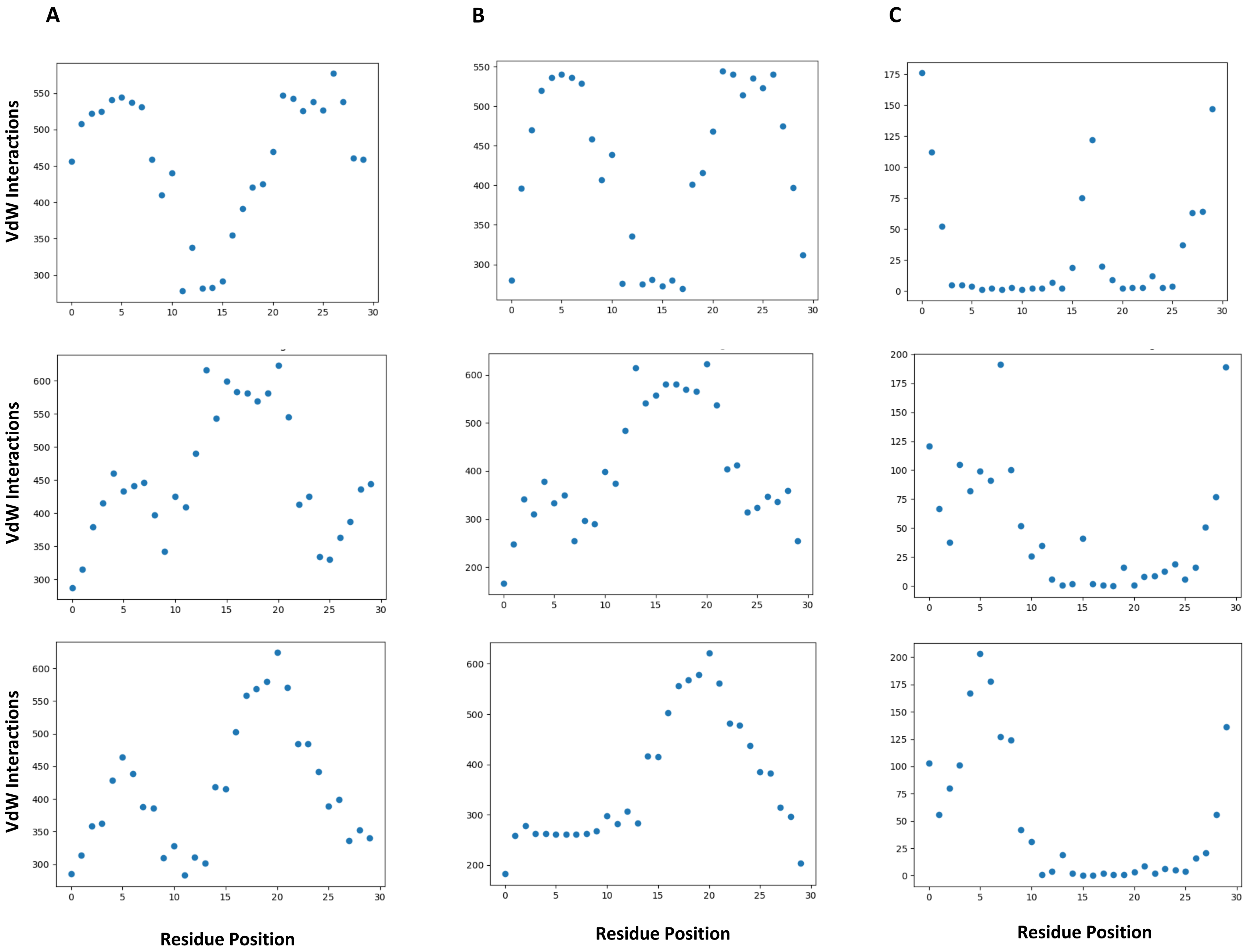
A
B
C
VdW Interactions
VdW Interactions
VdW Interactions
Residue Position
Residue Position
Residue Position
