# Supplementary material for: Deriving and Using Descriptors of Elementary Functions in Rational Protein Design
Source: Front Bioinform. 2021 Apr 13;1:657529. doi: 10.3389/fbinf.2021.657529 (PMC9581014; doi:10.3389/fbinf.2021.657529)
Supplement: Supplementary file 5 [file Image_3.PDF]

Figure S3. Isotropic hydrogen bond distributions within 3.5A per residue position where the residue acts as an acceptor, for DxDxD, GxGxxG, and GxxGxG signatures (in rows 1-3, respectively) of corresponding elementary functions. See main text for designation of elementary functions. The first column (A) indicates the total number of bonds, the second (B) are for bonds where the other party is within the length-30 loop, and the third (C) are for bonds where the other party is outside of the loop.

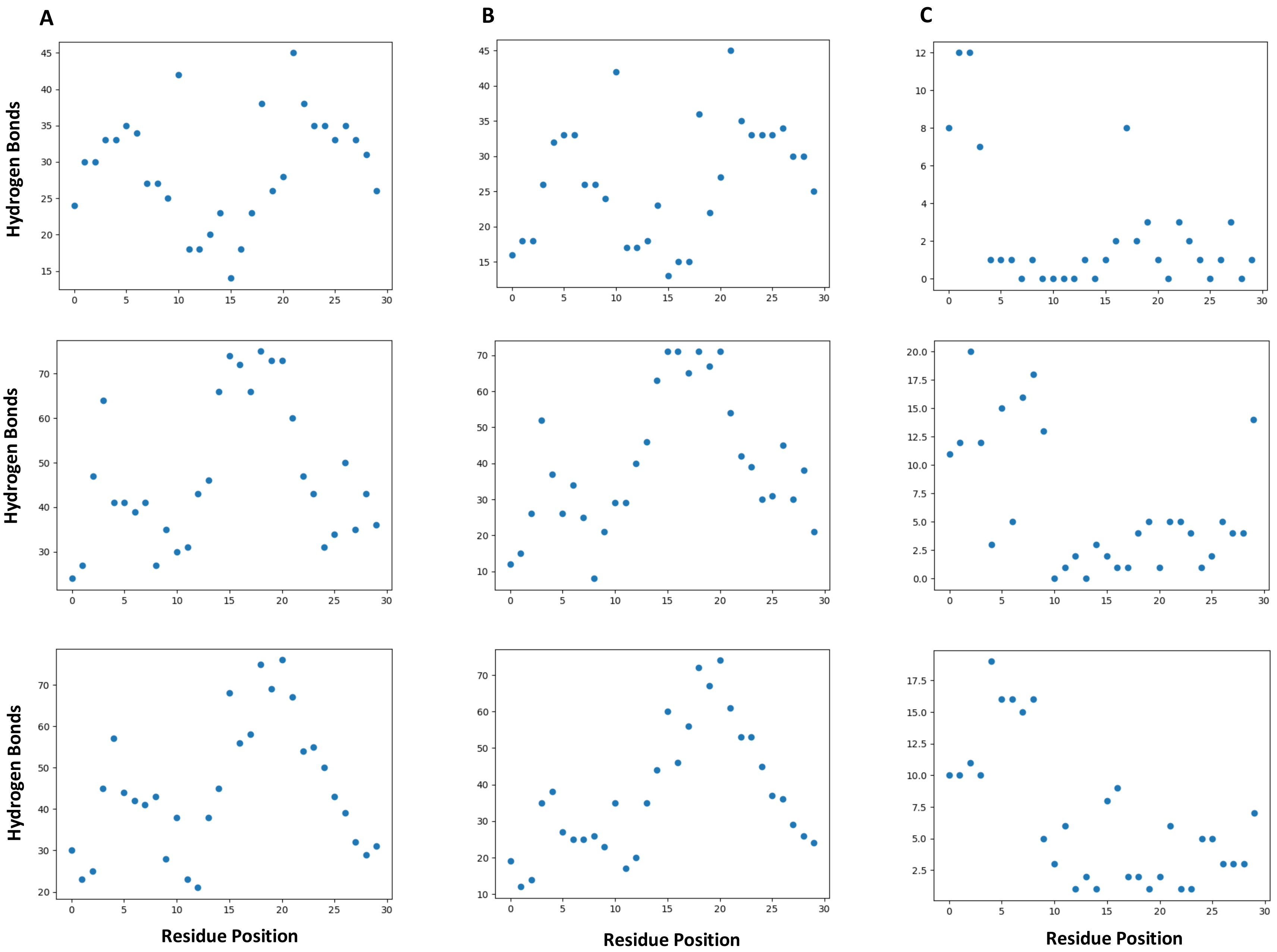
A
B
C
Hydrogen Bonds
Hydrogen Bonds
Hydrogen Bonds
Residue Position
Residue Position
Residue Position
