# Supplementary material for: Deriving and Using Descriptors of Elementary Functions in Rational Protein Design
Source: Front Bioinform. 2021 Apr 13;1:657529. doi: 10.3389/fbinf.2021.657529 (PMC9581014; doi:10.3389/fbinf.2021.657529)
Supplement: Supplementary file 6 [file Image_4.PDF]

Figure S4. Isotropic hydrogen bond distributions within 3.5A per residue position where the residue acts as a donor, for DxDxD, GxGxxG, and GxxGxG signatures (in rows 1-3, respectively) of corresponding elementary functions. The first column (A) indicates the total number of bonds, the second (B) are for bonds where the other party is within the length-30 loop, and the third (C) are for bonds where the other party is outside of the loop.

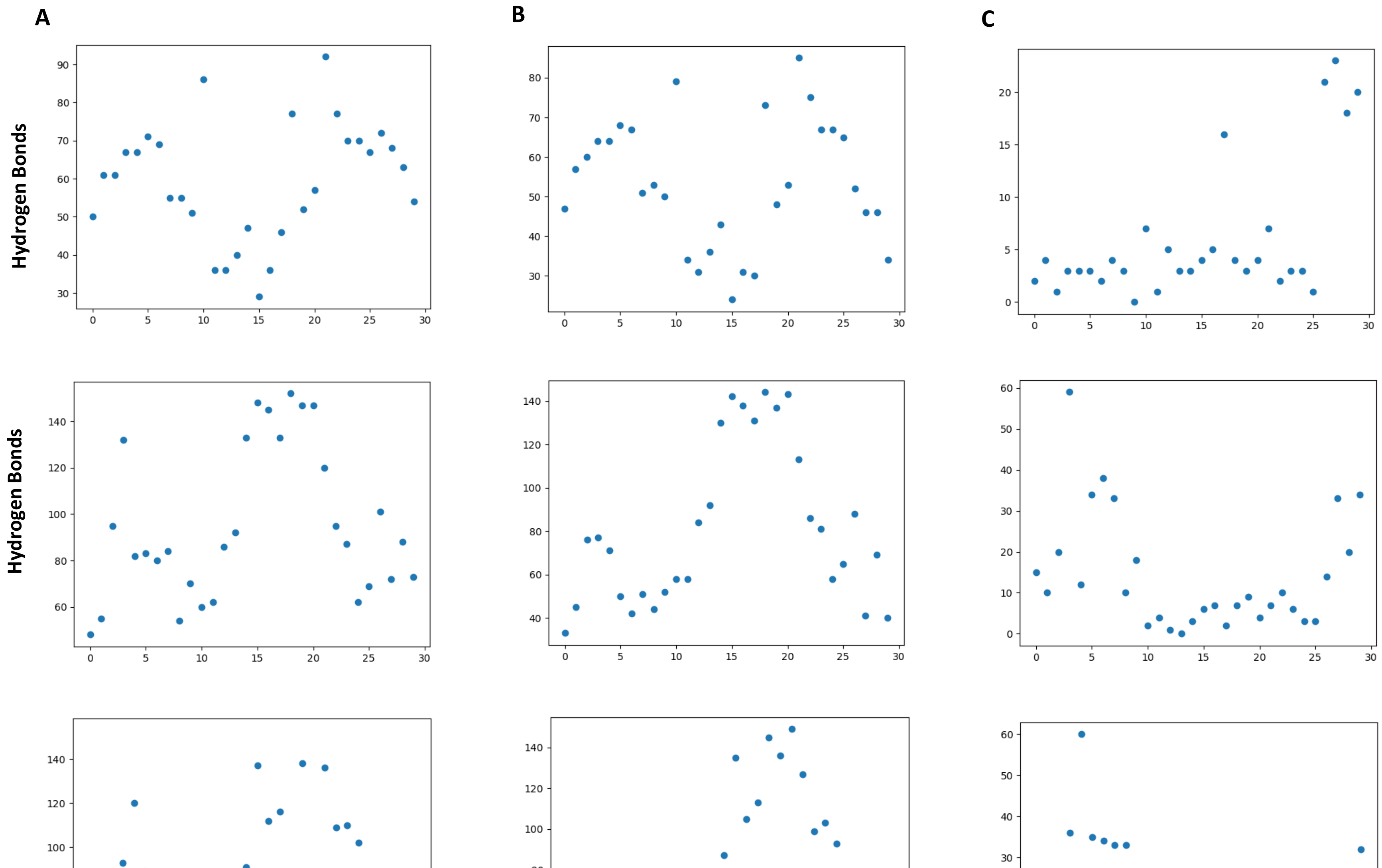

Hydrogen Bonds

Residue Position

Residue Position

Residue Position
