# Supplementary material for: Deriving and Using Descriptors of Elementary Functions in Rational Protein Design
Source: Front Bioinform. 2021 Apr 13;1:657529. doi: 10.3389/fbinf.2021.657529 (PMC9581014; doi:10.3389/fbinf.2021.657529)
Supplement: Supplementary file 7 [file Image_5.PDF]

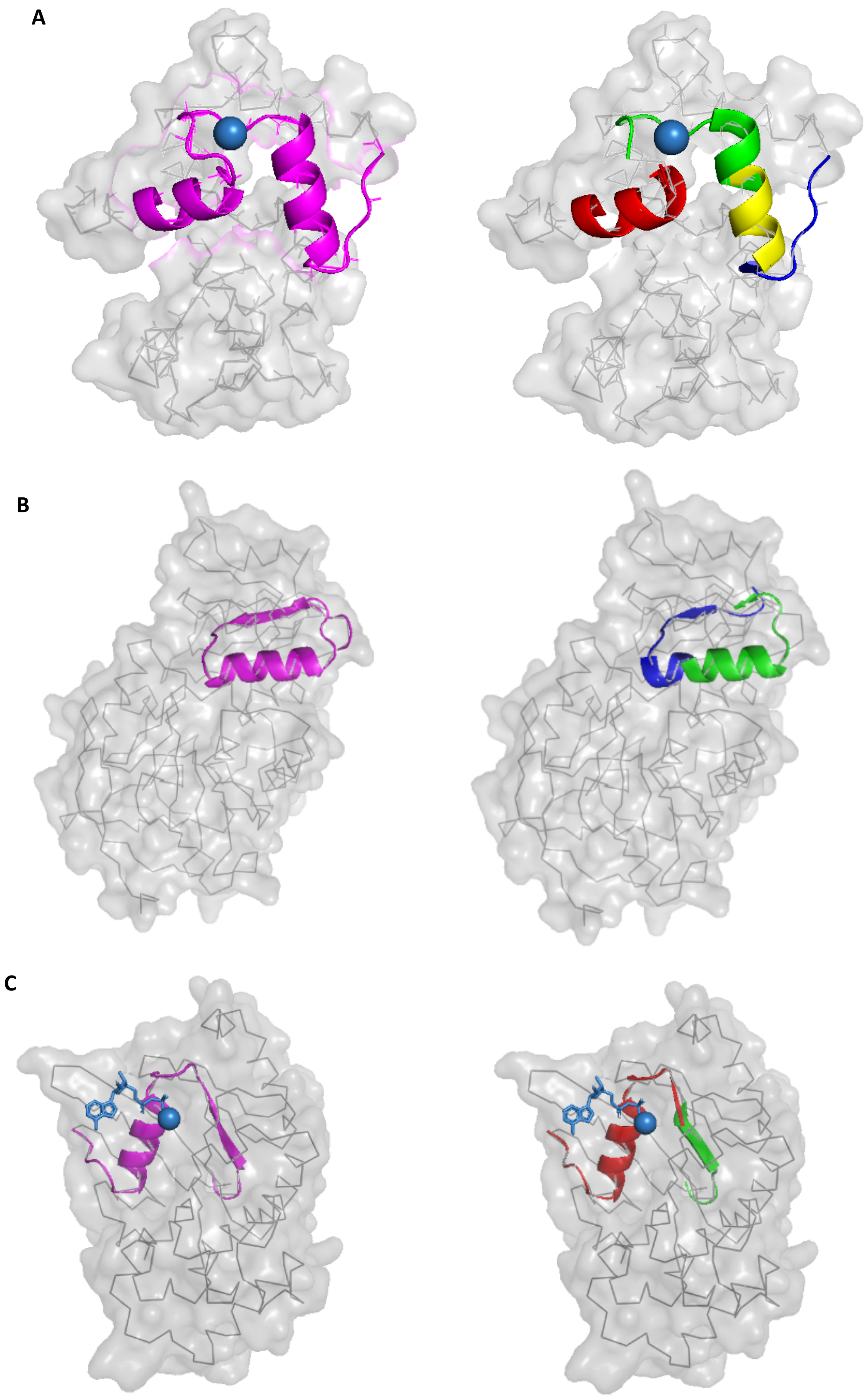
A
B
C

Figure S5. Representative structures with labelled loops and their recombinant realizations. The structures 1A29 representing the EF-hand loop (A), 1A71 representing the phosphate-binding motif in dinucleotide containing ligand (B), and 1G6H representing the P-loop (C), are used to find the best recombinant substructure to fit the original functional loop. Ca2+ ions are present in the EF-hand loop (A), while ADP and MG2+ are in the P-loop (C). The purple-marked loops in the left column are original elementary functional units that had to be replaced, structures in the right column contain new loops consisting of segments of different structures that provide the best fit to the original loop. Assessment of fitness is done by using a proxy metric that approximates the likelihood of the original structure belonging to the descriptor distributions. This is obtained by first training clustering models on the descriptor feature distributions, with each feature having their own distinct model to fit the idiosyncrasies of the features. For instance, dihedral torsional angles are mapped onto a Ramachandran plot and clustered using the EM algorithm, with some pre- and post-processing done such that a query of the EM model with a given structure at a specific residue will return the likelihood of the point belonging to the distribution, weighted by the degree of clustering in the torsional distribution relative to other structural features at that position. These weighted scores are summed for every feature and ranked, with top fits returned per residue position. This information can be useful, for example, if one needs to fit only a specific segment of the functional loop. For replacements involving the entire functional loop as a single entity, DEFINED-PROTEINS also return the structure with the best fit summed across all residue positions, weighted according to how important the fit at each position is assessed to be for the stability of the overall structure, based on their degree of conservation. Finally, in cases where a recombinant segment can potentially be used, the program identifies the best possible fit by merging a few structures in the descriptor into a single entity. Excessively short additions that are not practical in reality are disallowed as well.
