# Supplementary material for: Deriving and Using Descriptors of Elementary Functions in Rational Protein Design
Source: Front Bioinform. 2021 Apr 13;1:657529. doi: 10.3389/fbinf.2021.657529 (PMC9581014; doi:10.3389/fbinf.2021.657529)

Figure S6. Example of the output of the diphosphate-binding descriptor realization in the horse liver alcohol dehydrogenase (PDB ID: 1A71). The score plot (middle) shows the per-residue fitting score for the segments that provide the best replacement in the structure (top), with the descriptor signature logo below

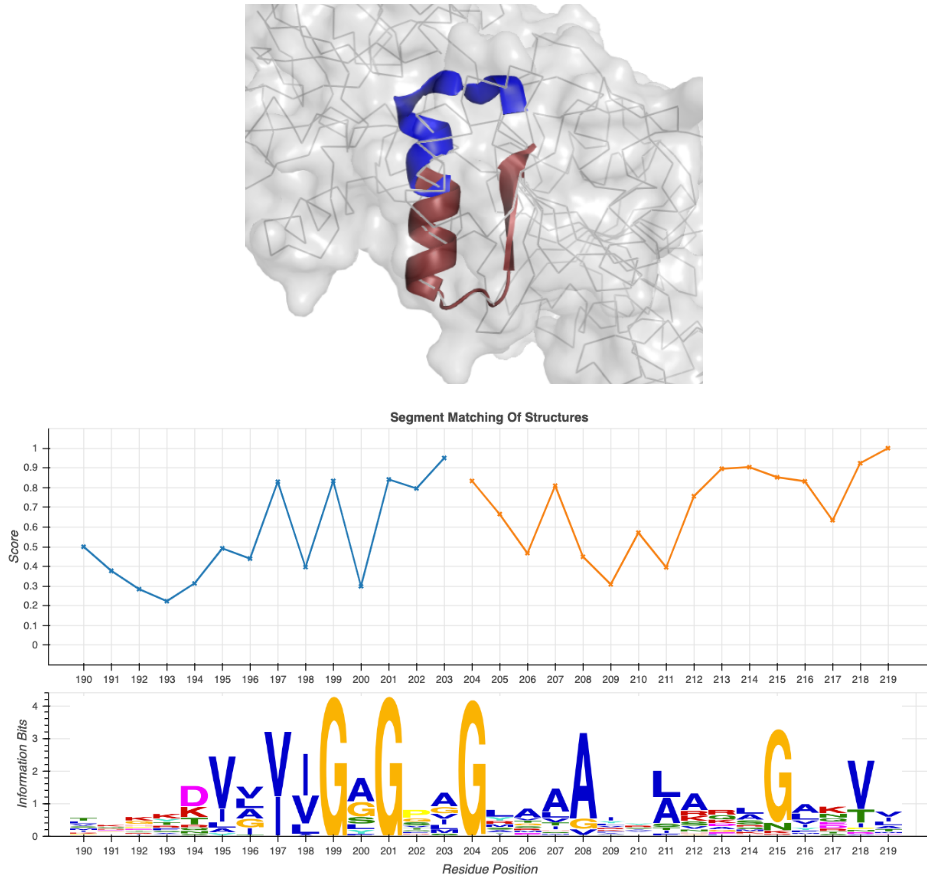

Supplement: Supplementary file 8 [file Image_6.PDF]
