# Supplementary material for: Deriving and Using Descriptors of Elementary Functions in Rational Protein Design
Source: Front Bioinform. 2021 Apr 13;1:657529. doi: 10.3389/fbinf.2021.657529 (PMC9581014; doi:10.3389/fbinf.2021.657529)

Figure S7. Single-structure replacements for the phosphate binding in dinucleotide-containing ligands (GxGxxG) using descriptors of the phosphate binding in nucleotide-containing ligands (GxxGxG). (A-C) Grafting of the phosphate-binding signature in dinucleotide-containing ligands (GxGxxG) in proteins with P-loop (GxxGxG) elementary function. Single-loop realizations of descriptors are shown in proteins with PDB IDs: 1H5Y, 1BWV, and 1O5K. (D-F) Single-loop realizations of the descriptor of P-loop (GxxGxG) elementary function in proteins (1SKY, 1II2, and 1NI3, respectively) with elementary functional loops of the phosphate-binding in dinucleotide-containing ligands (GxGxxG). The original structures are in green.

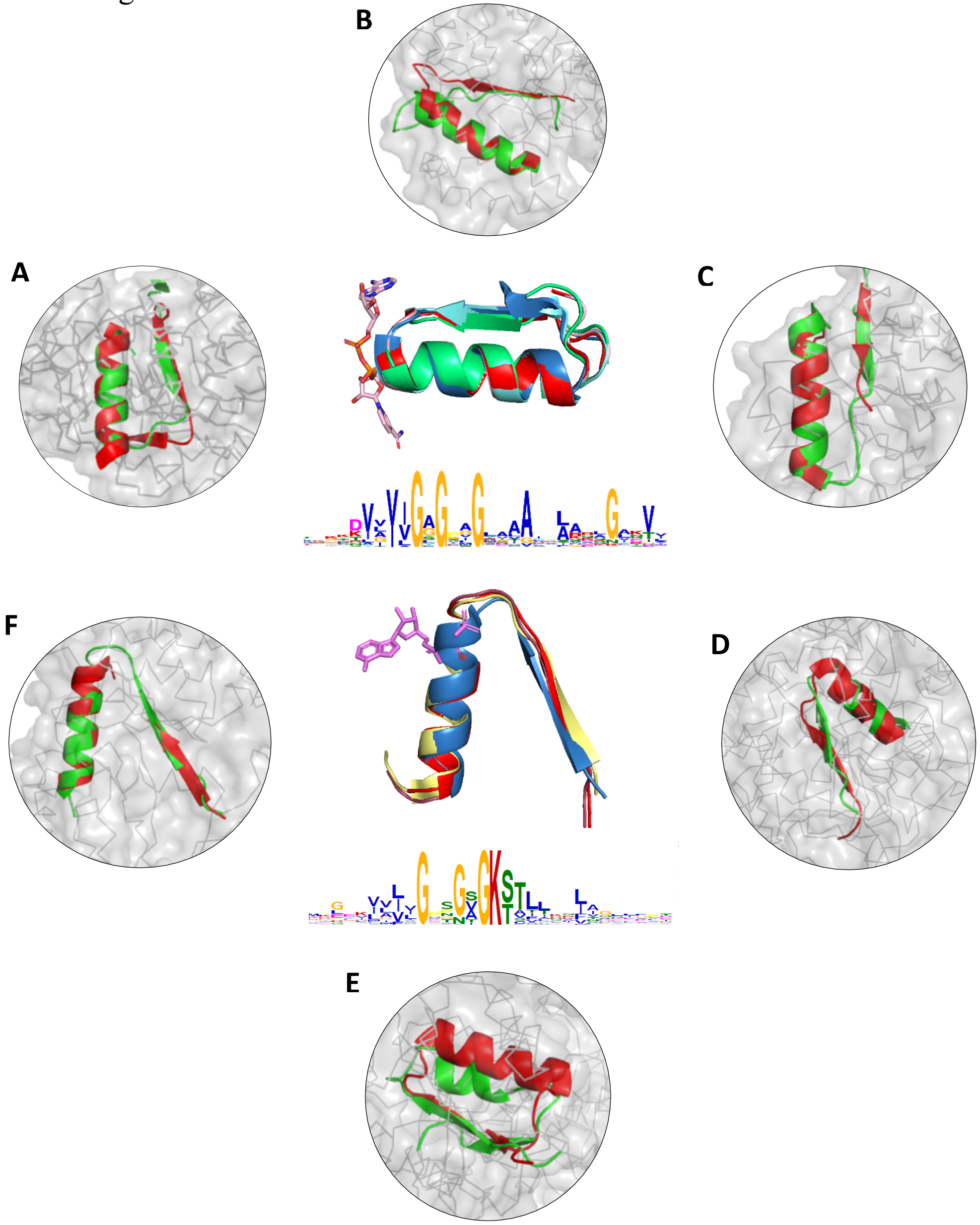

Supplement: Supplementary file 9 [file Image_7.PDF]
