# Supplementary material for: Deriving and Using Descriptors of Elementary Functions in Rational Protein Design
Source: Front Bioinform. 2021 Apr 13;1:657529. doi: 10.3389/fbinf.2021.657529 (PMC9581014; doi:10.3389/fbinf.2021.657529)

Figure S8. Results of the single-loop cross-grafting of the phosphate binding in dinucleotide (GxGxxG) and nucleotide-containing (GxxGxG) ligands in form of alignments. Replacement of the P-loop elementary function in 1NI3 (A) and of the phosphate-binding signature in dinucleotide-containing ligands in 1BWV(B). The thick, thin, and arrow sections represent the beta sheet, turn section, and alpha helix secondary structures respectively.

1NI3	G	N	N	L	K	T	G	I	V	G	M	P	N	V	G	K	S	T	F	F	R	A	I	T	K	S	V	L	G	N
4Q3N	M	R	N	S	K	V	V	V	V	G	T	G	F	V	G	M	S	Y	A	Y	A	M	V	N	Q	G	T	V	E	E
Score	0.46	0.25	0.87	0.30	0.78	0.54	0.68	0.60	0.80	0.85	0.40	0.72	0.32	0.77	0.78	0.34	0.65	0.55	0.40	0.82	0.40	0.38	0.70	0.23	0.22	0.32	0.45	0.42	0.50	0.40

1NI3
4Q3N

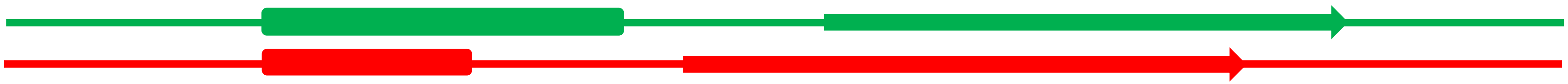

1BWV	G	E	D	V	V	L	Q	F	G	G	G	T	I	G	H	P	D	G	I	Q	A	G	A	T	A	N	R	V	A	L
3JBH	E	N	Q	S	M	L	I	T	G	E	S	G	A	G	K	T	E	N	T	K	K	V	I	A	Y	Y	A	N	V	G
Score	0.40	0.40	0.62	0.70	0.60	0.75	0.23	0.20	0.95	0.52	0.84	0.76	0.30	0.80	0.40	0.60	0.50	0.45	0.60	0.72	0.70	0.15	0.60	0.65	0.75	0.60	0.50	0.40	0.80	0.72

1BWV
3JBH

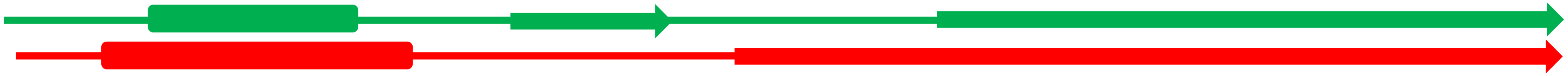

Supplement: Supplementary file 10 [file Image_8.PDF]
